# Crystal structure of 5,10,15-triphenyl-20-(4,4,5,5-tetra­methyl-1,3,2-dioxaborolan-2-yl)porphyrin

**DOI:** 10.1107/S1600536814019680

**Published:** 2014-09-06

**Authors:** Mathias O. Senge, Hans-Georg Eckhardt

**Affiliations:** a SFI Tetrapyrrole Laboratory, School of Chemistry, Trinity College Dublin, Dublin 2, Ireland

**Keywords:** crystal structure, porphyrinoid, tetra­pyrroles, porphyrins

## Abstract

In the title compound, C_44_H_37_BN_4_O_2_, the dihedral angle between the plane of the porphyrin macrocycle ring system [r.m.s. deviation = 0.159 (1) Å] and those of three phenyl rings are 66.11 (4), 74.75 (4) and 57.00 (4)°. The conformational distortion is characterized by a mixture of ruffled, saddle and in-plane distortion modes. In the crystal, the porphyrin mol­ecules are linked by C—H⋯π inter­actions into supra­molecular chains running along the *a-*axis direction. A pair of bifurcated N—H⋯(N,N) hydrogen bonds occur across the central region of the macrocycle.

## Related literature   

For the structure and conformation of porphyrins, see: Scheidt & Lee (1987 ▶); Jentzen *et al.* (1997 ▶); Senge (2000 ▶, 2006 ▶). For the synthesis, see: Finnigan *et al.* (2011 ▶). For the handling of crystals, see Hope (1994 ▶). For related boronyl porphyrin structures, see: Hyslop *et al.* (1998 ▶); Schwalbe *et al.* (2012 ▶). For other recent free base porphyrin structures, see: Miranda *et al.* (2012 ▶); Leonarska *et al.* (2012 ▶); Senge (2013 ▶).
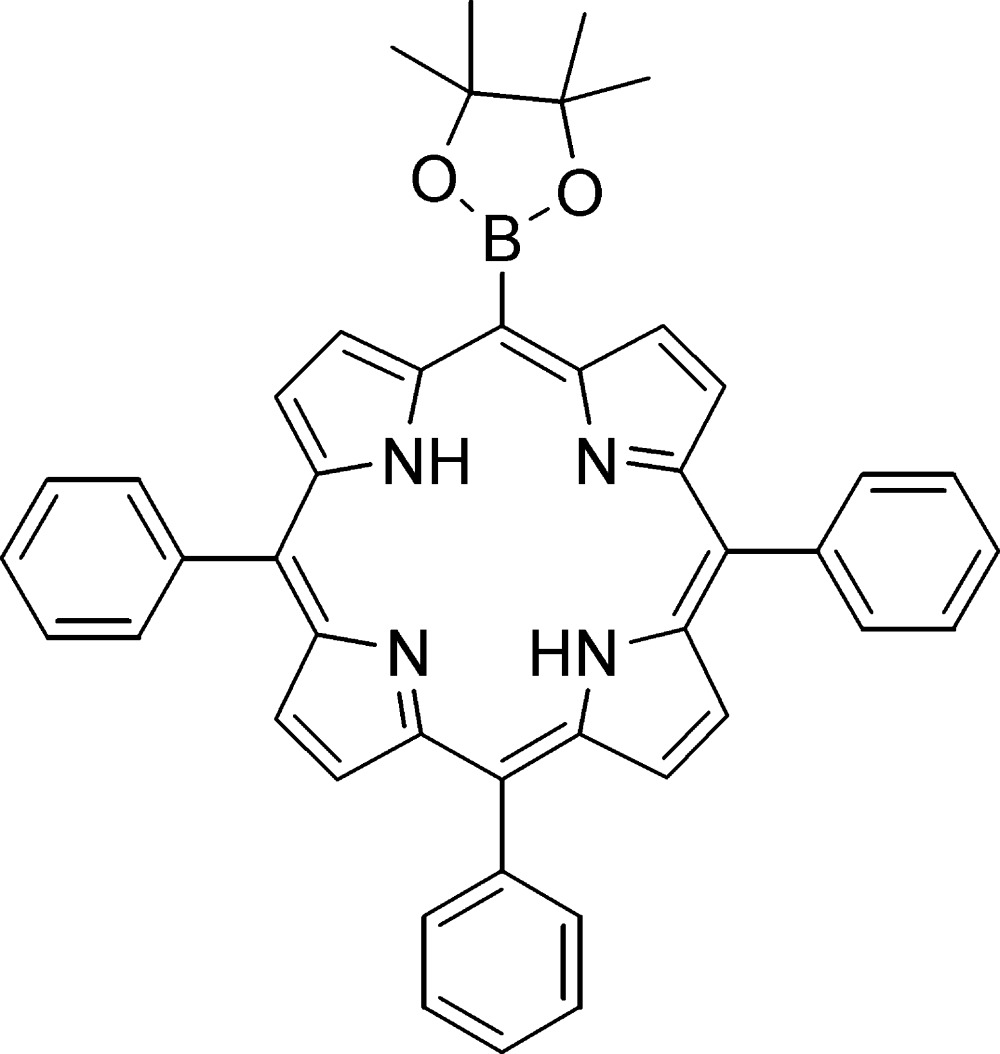


## Experimental   

### Crystal data  

C_44_H_37_BN_4_O_2_
*M* *_r_* = 664.59
Monoclinic, 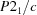

*a* = 13.0758 (5) Å
*b* = 10.5245 (4) Å
*c* = 27.1358 (10) Å
β = 110.353 (2)°
*V* = 3501.2 (2) Å^3^
*Z* = 4
Cu *K*α radiation
μ = 0.61 mm^−1^
*T* = 100 K
0.50 × 0.50 × 0.35 mm

### Data collection  

Bruker APEXII CCD diffractometer
Absorption correction: multi-scan (*SADABS*; Bruker, 2005 ▶) *T*_min_ = 0.751, *T*_max_ = 0.815
25108 measured reflections
5993 independent reflections
5788 reflections with *I* > 2σ(*I*)
*R*_int_ = 0.029

### Refinement  

*R*[*F* ^2^ > 2σ(*F* ^2^)] = 0.041
*wR*(*F* ^2^) = 0.103
*S* = 1.03
5993 reflections
464 parameters
H-atom parameters constrained
Δρ_max_ = 0.27 e Å^−3^
Δρ_min_ = −0.27 e Å^−3^

### 

Data collection: *APEX2* (Bruker, 2005 ▶); cell refinement: *SAINT* (Bruker, 2005 ▶); data reduction: *SAINT*; program(s) used to solve structure: *SHELXS97* (Sheldrick, 2008 ▶); program(s) used to refine structure: *SHELXL97* (Sheldrick, 2008 ▶); molecular graphics: *XP* in *SHELXTL* (Sheldrick, 2008 ▶) and *DIAMOND* (Brandenburg, 1998 ▶); software used to prepare material for publication: *SHELXTL-Plus* (Sheldrick, 2008 ▶).

## Supplementary Material

Crystal structure: contains datablock(s) I, New_Global_Publ_Block. DOI: 10.1107/S1600536814019680/lx2293sup1.cif

Structure factors: contains datablock(s) I. DOI: 10.1107/S1600536814019680/lx2293Isup2.hkl

**Fig. 1.**
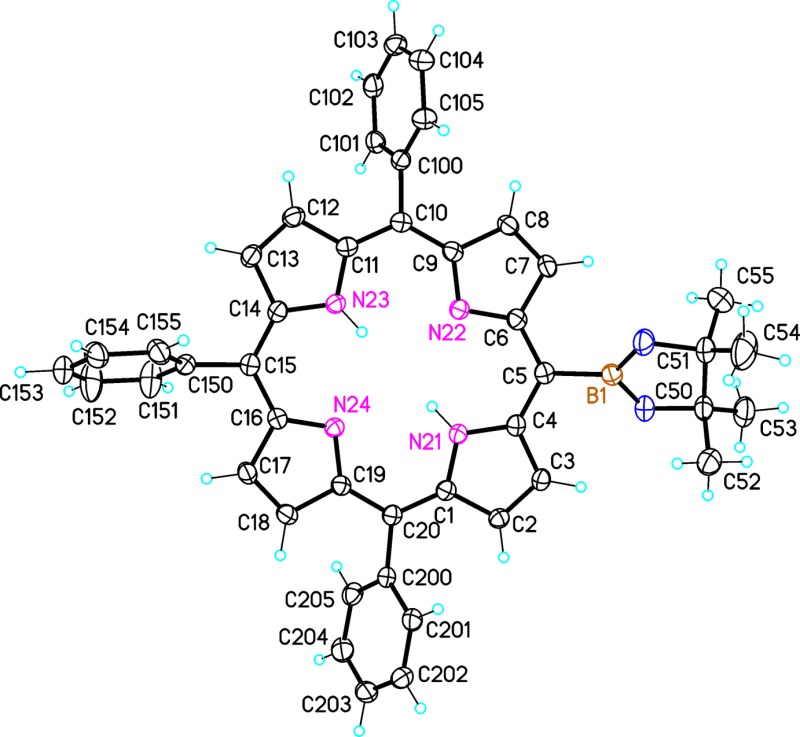
Molecular structure of the title compound. Thermal ellipsoids are drawn at 50% probability level.

**Fig. 2.**
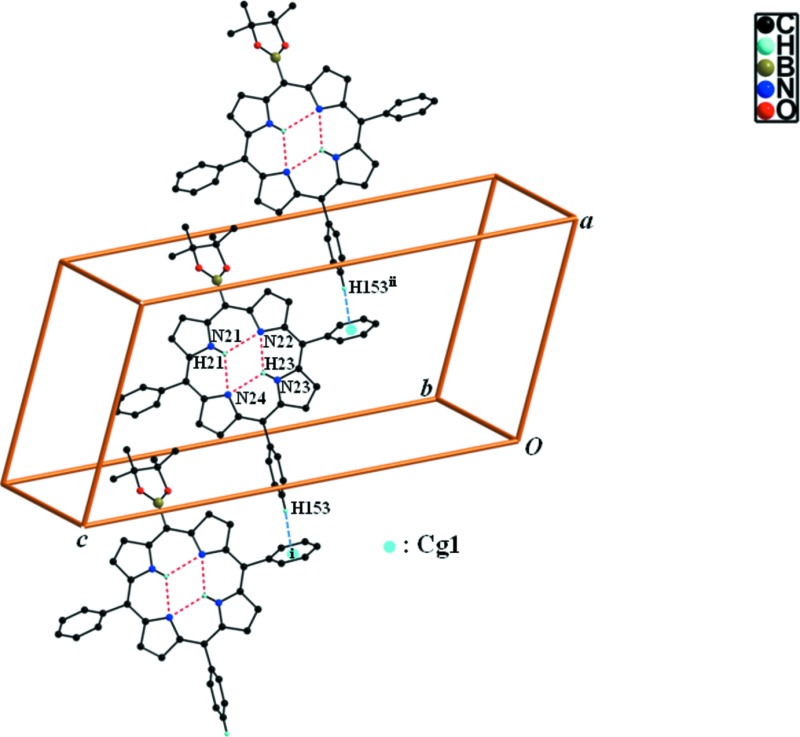
A view of the N—H···N and C—H···π interactions (dotted lines) in the crystal structure of the title compound. H atoms non-participating in hydrogen-bonding were omitted for clarity. [Symmetry code: (i) x -1, y, z; (ii) x + 1, y, z.]

. DOI: 10.1107/S1600536814019680/lx2293fig1.tif
Mol­ecular structure of the title compound. Thermal ellipsoids are drawn at 50% probability level.

x y z x y z . DOI: 10.1107/S1600536814019680/lx2293fig2.tif
A view of the N—H⋯N and C—H⋯π inter­actions (dotted lines) in the crystal structure of the title compound. H atoms non-participating in hydrogen-bonding were omitted for clarity. [Symmetry code: (i) *x* − 1, *y*, *z*; (ii) *x* + 1, *y*, *z*.]

CCDC reference: 1022040

Additional supporting information:  crystallographic information; 3D view; checkCIF report

## Figures and Tables

**Table 1:** Hydrogen-bond geometry (Å, °) *Cg*1 is the centroid of the C100–C105 phenyl ring.

*D*—H⋯*A*	*D*—H	H⋯*A*	*D*⋯*A*	*D*—H⋯*A*
N21—H21⋯N24	0.88	2.35	2.9084 (16)	122
N21—H21⋯N22	0.88	2.38	2.9266 (16)	121
N23—H23⋯N24	0.88	2.34	2.8978 (16)	121
N23—H23⋯N22	0.88	2.35	2.8986 (16)	120
C153—H153⋯*Cg*1^i^	0.95	2.58	3.488 (2)	160

Symmetry code: (i) 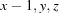.

## supplementary crystallographic information

### S1. Comment

The compound was prepared *via* borylation of 5-bromo-10,15,20-triphenylporphyrin with 4,4,5,5-tetramethyl-1,3,2-dioxaborolane under dichlorobis(triphenylphosphine)palladium(II) catalysis (Finnigan *et al.*, 2011). Crystallization from CH_2_Cl_2_/CH_3_OH yielded monoclinic crystals without solvent inclusion.

The compound was investigated with regard to its macrocycle conformation. The porphyrin macrocycle ring system, with a mean deviation of 0.159 (1) Å from the least-squares plane defined by the 24 constituent atoms. Three phenyl rings are essentially planar, with a mean deviation of 0.001 (1) (C100–C105), 0.003 (1) (C150–C155) and 0.007 (1) (C200–C205) Å from the least-squares plane defined by the six constituent atoms. The dihedral angle formed by the porphyrin macrocycle ring system and three phenyl rings are 66.11 (4)(C100–C105), 74.75 (4) (C150–C155) and 57.00 (4) (C200–C205) °, respectively. A conformational analysis was performed using the NSD (normal structural decomposition) method developed by Shelnutt and coworkers (Jentzen *et al.*, 1997). The compound exhibits a moderate degree of conformational distortion. The main contributing out-of-plane distortion modes are *ruf*, *sad* and *wav*. Likewise moderate contributions from macrocycle breathing and *N-str* are observed for the in-plane distortions. Related Zn(II) complexes, *e.g.* {5,15-diphenyl-10-(4,4,5,5-tetramethyl-1,3,2-dioxaborolan-2-yl)porphyrinato}zinc(II) (Hyslop *et al.*, 1998; Schwalbe *et al.*, 2012) exhibit more planar conformations. Other recent porphyrin free base structures have been reported by Miranda *et al.* (2012), Leonarska *et al.* (2012), and Senge (2013).

In the crystal packing (Fig. 2), the porphyrin molecules are linked by C—H···π interactions (Table 1, Cg1 is the centroid of the C100–C105 phenyl ring) into supramolecular chains running along the *a* axis. Also intramolecular N—H···N hydrogen bonds occur (Table 1).

### S2. Experimental

The title compound was prepared as described by Finnigan *et al.* (2011).

### Crystal data

**Table:**

C_44_H_37_BN_4_O_2_	*F*(000) = 1400
*M**_r_* = 664.59	*D*_x_ = 1.261 Mg m^−^^3^
Monoclinic, *P*2_1_/*c*	Cu *K*α radiation, λ = 1.54178 Å
Hall symbol: -P 2ybc	Cell parameters from 9566 reflections
*a* = 13.0758 (5) Å	θ = 4.1–66.8°
*b* = 10.5245 (4) Å	µ = 0.61 mm^−^^1^
*c* = 27.1358 (10) Å	*T* = 100 K
β = 110.353 (2)°	Triangle, purple
*V* = 3501.2 (2) Å^3^	0.50 × 0.50 × 0.35 mm
*Z* = 4	

### Data collection

**Table:**

Bruker APEXII CCD diffractometer	5993 independent reflections
Radiation source: sealed tube	5788 reflections with *I* > 2σ(*I*)
Graphite monochromator	*R*_int_ = 0.029
Detector resolution: 8.33 pixels mm^-1^	θ_max_ = 66.8°, θ_min_ = 4.0°
φ and ω scans	*h* = −15→15
Absorption correction: multi-scan (*SADABS*; Bruker, 2005)	*k* = −8→12
*T*_min_ = 0.751, *T*_max_ = 0.815	*l* = −31→32
25108 measured reflections	

### Refinement

**Table:**

Refinement on *F*^2^	Primary atom site location: structure-invariant direct methods
Least-squares matrix: full	Secondary atom site location: difference Fourier map
*R*[*F*^2^ > 2σ(*F*^2^)] = 0.041	Hydrogen site location: difference Fourier map
*wR*(*F*^2^) = 0.103	H-atom parameters constrained
*S* = 1.03	*w* = 1/[σ^2^(*F*_o_^2^) + (0.0402*P*)^2^ + 2.0737*P*] where *P* = (*F*_o_^2^ + 2*F*_c_^2^)/3
5993 reflections	(Δ/σ)_max_ < 0.001
464 parameters	Δρ_max_ = 0.27 e Å^−^^3^
0 restraints	Δρ_min_ = −0.27 e Å^−^^3^

### Special details

**Table:**

Geometry. All esds (except the esd in the dihedral angle between two l.s. planes) are estimated using the full covariance matrix. The cell esds are taken into account individually in the estimation of esds in distances, angles and torsion angles; correlations between esds in cell parameters are only used when they are defined by crystal symmetry. An approximate (isotropic) treatment of cell esds is used for estimating esds involving l.s. planes.
Refinement. Refinement of F^2^ against ALL reflections. The weighted R-factor wR and goodness of fit S are based on F^2^, conventional R-factors R are based on F, with F set to zero for negative F^2^. The threshold expression of F^2^ > 2sigma(F^2^) is used only for calculating R-factors(gt) etc. and is not relevant to the choice of reflections for refinement. R-factors based on F^2^ are statistically about twice as large as those based on F, and R- factors based on ALL data will be even larger. The carbon atoms of one phenyl unit show some degree of thermal librational movement.

### Fractional atomic coordinates and isotropic or equivalent isotropic displacement parameters (Å^2^)

**Table:**

	*x*	*y*	*z*	*U*_iso_*/*U*_eq_	
C1	0.55291 (11)	0.54316 (12)	0.72969 (5)	0.0213 (3)	
C2	0.63876 (11)	0.60493 (13)	0.77064 (5)	0.0229 (3)	
H2	0.6384	0.6249	0.8047	0.027*	
C3	0.72077 (11)	0.62999 (13)	0.75218 (5)	0.0233 (3)	
H3	0.7872	0.6721	0.7709	0.028*	
C4	0.68981 (11)	0.58188 (12)	0.69939 (5)	0.0209 (3)	
C5	0.75153 (11)	0.58579 (13)	0.66621 (5)	0.0221 (3)	
C6	0.72497 (11)	0.51900 (13)	0.61822 (5)	0.0225 (3)	
C7	0.80206 (12)	0.50156 (14)	0.59095 (6)	0.0281 (3)	
H7	0.8718	0.5401	0.5997	0.034*	
C8	0.75622 (12)	0.42099 (14)	0.55110 (6)	0.0280 (3)	
H8	0.7883	0.3894	0.5270	0.034*	
C9	0.64820 (11)	0.39125 (13)	0.55173 (5)	0.0223 (3)	
C10	0.57290 (11)	0.31342 (13)	0.51484 (5)	0.0219 (3)	
C11	0.46626 (11)	0.28910 (13)	0.51343 (5)	0.0221 (3)	
C12	0.38183 (12)	0.22656 (15)	0.47235 (6)	0.0278 (3)	
H12	0.3891	0.1897	0.4418	0.033*	
C13	0.28955 (12)	0.22871 (14)	0.48442 (6)	0.0280 (3)	
H13	0.2207	0.1947	0.4637	0.034*	
C14	0.31440 (11)	0.29137 (13)	0.53390 (5)	0.0222 (3)	
C15	0.24417 (11)	0.31216 (13)	0.56185 (5)	0.0226 (3)	
C16	0.27360 (11)	0.36879 (13)	0.61174 (5)	0.0222 (3)	
C17	0.19703 (11)	0.39354 (15)	0.63825 (6)	0.0269 (3)	
H17	0.1209	0.3767	0.6252	0.032*	
C18	0.25478 (11)	0.44510 (15)	0.68516 (6)	0.0265 (3)	
H18	0.2272	0.4709	0.7117	0.032*	
C19	0.36661 (11)	0.45374 (13)	0.68739 (5)	0.0213 (3)	
C20	0.45034 (11)	0.50854 (13)	0.73035 (5)	0.0212 (3)	
N21	0.58829 (9)	0.52924 (11)	0.68782 (4)	0.0211 (2)	
H21	0.5511	0.4919	0.6579	0.025*	
N22	0.63209 (9)	0.45164 (11)	0.59351 (4)	0.0214 (2)	
N23	0.42208 (9)	0.32698 (11)	0.54992 (4)	0.0213 (2)	
H23	0.4573	0.3679	0.5792	0.026*	
N24	0.37618 (9)	0.40825 (11)	0.64213 (4)	0.0209 (2)	
B1	0.85661 (13)	0.67155 (15)	0.68378 (6)	0.0242 (3)	
C50	0.99982 (12)	0.78798 (15)	0.73694 (6)	0.0314 (3)	
C51	0.99019 (14)	0.80462 (17)	0.67871 (7)	0.0377 (4)	
C52	0.94995 (14)	0.89499 (17)	0.75792 (7)	0.0405 (4)	
H52A	0.8746	0.9086	0.7345	0.061*	
H52B	0.9922	0.9729	0.7598	0.061*	
H52C	0.9507	0.8731	0.7931	0.061*	
C53	1.11315 (13)	0.75979 (18)	0.77465 (8)	0.0426 (4)	
H53A	1.1116	0.7523	0.8104	0.064*	
H53B	1.1624	0.8289	0.7734	0.064*	
H53C	1.1390	0.6799	0.7646	0.064*	
C54	0.9911 (2)	0.9413 (2)	0.66153 (9)	0.0655 (7)	
H54A	0.9874	0.9436	0.6248	0.098*	
H54B	1.0585	0.9826	0.6838	0.098*	
H54C	0.9281	0.9862	0.6648	0.098*	
C55	1.07129 (17)	0.7236 (3)	0.66391 (9)	0.0624 (6)	
H55A	1.0661	0.6352	0.6742	0.094*	
H55B	1.1453	0.7553	0.6821	0.094*	
H55C	1.0547	0.7280	0.6258	0.094*	
C100	0.60355 (11)	0.25149 (13)	0.47213 (5)	0.0227 (3)	
C101	0.60723 (11)	0.11908 (14)	0.46904 (6)	0.0260 (3)	
H101	0.5935	0.0686	0.4951	0.031*	
C102	0.63066 (12)	0.06045 (14)	0.42833 (6)	0.0291 (3)	
H102	0.6330	−0.0296	0.4267	0.035*	
C103	0.65068 (12)	0.13354 (15)	0.39013 (6)	0.0300 (3)	
H103	0.6664	0.0936	0.3622	0.036*	
C104	0.64771 (12)	0.26504 (15)	0.39283 (6)	0.0293 (3)	
H104	0.6617	0.3152	0.3668	0.035*	
C105	0.62430 (12)	0.32356 (14)	0.43358 (6)	0.0269 (3)	
H105	0.6224	0.4137	0.4352	0.032*	
C150	0.12827 (11)	0.27037 (14)	0.53697 (5)	0.0254 (3)	
C151	0.09046 (14)	0.16336 (18)	0.55491 (8)	0.0473 (5)	
H151	0.1392	0.1135	0.5822	0.057*	
C152	−0.01833 (16)	0.1288 (2)	0.53314 (9)	0.0565 (6)	
H152	−0.0434	0.0548	0.5455	0.068*	
C153	−0.09030 (13)	0.20004 (18)	0.49394 (7)	0.0399 (4)	
H153	−0.1648	0.1759	0.4794	0.048*	
C154	−0.05394 (12)	0.30615 (18)	0.47587 (6)	0.0374 (4)	
H154	−0.1034	0.3560	0.4488	0.045*	
C155	0.05526 (12)	0.34111 (17)	0.49711 (6)	0.0326 (4)	
H155	0.0800	0.4143	0.4841	0.039*	
C200	0.42271 (11)	0.53497 (14)	0.77821 (5)	0.0227 (3)	
C201	0.42174 (12)	0.65766 (15)	0.79739 (6)	0.0284 (3)	
H201	0.4450	0.7270	0.7815	0.034*	
C202	0.38703 (12)	0.67918 (17)	0.83956 (6)	0.0350 (4)	
H202	0.3871	0.7631	0.8525	0.042*	
C203	0.35234 (12)	0.57919 (18)	0.86278 (6)	0.0371 (4)	
H203	0.3275	0.5945	0.8912	0.044*	
C204	0.35386 (13)	0.45689 (17)	0.84452 (6)	0.0350 (4)	
H204	0.3302	0.3880	0.8605	0.042*	
C205	0.38975 (12)	0.43456 (15)	0.80300 (5)	0.0278 (3)	
H205	0.3920	0.3500	0.7912	0.033*	
O1	0.92954 (8)	0.67730 (10)	0.73411 (4)	0.0297 (2)	
O2	0.88183 (9)	0.75138 (11)	0.65026 (4)	0.0355 (3)	

### Atomic displacement parameters (Å^2^)

**Table:**

	*U*^11^	*U*^22^	*U*^33^	*U*^12^	*U*^13^	*U*^23^
C1	0.0237 (7)	0.0168 (6)	0.0236 (7)	0.0005 (5)	0.0084 (5)	0.0006 (5)
C2	0.0250 (7)	0.0209 (7)	0.0227 (7)	−0.0002 (6)	0.0082 (5)	−0.0009 (5)
C3	0.0218 (7)	0.0200 (7)	0.0265 (7)	−0.0019 (5)	0.0064 (5)	−0.0013 (6)
C4	0.0203 (6)	0.0155 (6)	0.0255 (7)	0.0011 (5)	0.0063 (5)	0.0010 (5)
C5	0.0220 (7)	0.0174 (7)	0.0267 (7)	0.0012 (5)	0.0083 (5)	0.0015 (5)
C6	0.0249 (7)	0.0176 (7)	0.0263 (7)	−0.0008 (5)	0.0106 (6)	0.0014 (5)
C7	0.0260 (7)	0.0281 (8)	0.0341 (8)	−0.0076 (6)	0.0155 (6)	−0.0038 (6)
C8	0.0297 (8)	0.0282 (8)	0.0322 (8)	−0.0045 (6)	0.0186 (6)	−0.0043 (6)
C9	0.0262 (7)	0.0178 (7)	0.0256 (7)	−0.0004 (5)	0.0122 (6)	0.0021 (5)
C10	0.0263 (7)	0.0177 (7)	0.0230 (7)	0.0018 (5)	0.0101 (6)	0.0020 (5)
C11	0.0252 (7)	0.0186 (7)	0.0229 (7)	0.0027 (5)	0.0087 (5)	0.0002 (5)
C12	0.0268 (7)	0.0308 (8)	0.0252 (7)	0.0019 (6)	0.0080 (6)	−0.0069 (6)
C13	0.0232 (7)	0.0301 (8)	0.0273 (7)	−0.0004 (6)	0.0047 (6)	−0.0075 (6)
C14	0.0208 (7)	0.0205 (7)	0.0233 (7)	0.0013 (5)	0.0050 (5)	−0.0006 (5)
C15	0.0213 (7)	0.0206 (7)	0.0243 (7)	0.0006 (5)	0.0058 (5)	0.0011 (5)
C16	0.0207 (6)	0.0225 (7)	0.0235 (7)	0.0000 (5)	0.0077 (5)	0.0015 (5)
C17	0.0198 (7)	0.0363 (8)	0.0252 (7)	−0.0029 (6)	0.0084 (6)	0.0001 (6)
C18	0.0238 (7)	0.0339 (8)	0.0240 (7)	−0.0008 (6)	0.0111 (6)	−0.0008 (6)
C19	0.0231 (7)	0.0194 (7)	0.0224 (7)	−0.0007 (5)	0.0091 (5)	0.0013 (5)
C20	0.0235 (7)	0.0168 (6)	0.0233 (7)	−0.0002 (5)	0.0083 (5)	0.0007 (5)
N21	0.0205 (6)	0.0200 (6)	0.0228 (6)	−0.0023 (5)	0.0074 (4)	−0.0024 (5)
N22	0.0237 (6)	0.0178 (6)	0.0234 (6)	−0.0011 (5)	0.0091 (5)	−0.0005 (5)
N23	0.0216 (6)	0.0212 (6)	0.0208 (5)	−0.0002 (5)	0.0070 (4)	−0.0022 (4)
N24	0.0216 (6)	0.0201 (6)	0.0208 (5)	−0.0004 (5)	0.0072 (4)	−0.0007 (4)
B1	0.0240 (8)	0.0198 (8)	0.0309 (8)	0.0013 (6)	0.0121 (7)	−0.0018 (6)
C50	0.0267 (7)	0.0292 (8)	0.0394 (9)	−0.0090 (6)	0.0130 (7)	−0.0062 (7)
C51	0.0372 (9)	0.0385 (9)	0.0403 (9)	−0.0189 (7)	0.0172 (7)	−0.0085 (7)
C52	0.0371 (9)	0.0374 (9)	0.0480 (10)	−0.0056 (7)	0.0161 (8)	−0.0108 (8)
C53	0.0287 (8)	0.0416 (10)	0.0528 (10)	−0.0074 (7)	0.0082 (8)	−0.0063 (8)
C54	0.0834 (16)	0.0572 (13)	0.0492 (11)	−0.0405 (12)	0.0147 (11)	0.0048 (10)
C55	0.0476 (11)	0.0894 (17)	0.0638 (13)	−0.0248 (11)	0.0364 (10)	−0.0324 (12)
C100	0.0206 (6)	0.0224 (7)	0.0245 (7)	0.0006 (5)	0.0071 (5)	−0.0021 (6)
C101	0.0238 (7)	0.0224 (7)	0.0331 (8)	−0.0011 (6)	0.0115 (6)	−0.0013 (6)
C102	0.0248 (7)	0.0221 (7)	0.0407 (8)	−0.0009 (6)	0.0116 (6)	−0.0074 (6)
C103	0.0252 (7)	0.0353 (9)	0.0295 (7)	0.0034 (6)	0.0096 (6)	−0.0091 (6)
C104	0.0313 (8)	0.0327 (8)	0.0253 (7)	0.0048 (6)	0.0116 (6)	0.0020 (6)
C105	0.0320 (8)	0.0226 (7)	0.0274 (7)	0.0047 (6)	0.0118 (6)	0.0003 (6)
C150	0.0218 (7)	0.0286 (8)	0.0251 (7)	−0.0006 (6)	0.0073 (6)	−0.0054 (6)
C151	0.0320 (9)	0.0420 (10)	0.0543 (11)	−0.0088 (8)	−0.0021 (8)	0.0141 (9)
C152	0.0385 (10)	0.0521 (12)	0.0673 (13)	−0.0192 (9)	0.0037 (9)	0.0144 (10)
C153	0.0233 (8)	0.0526 (11)	0.0400 (9)	−0.0075 (7)	0.0063 (7)	−0.0086 (8)
C154	0.0235 (8)	0.0570 (11)	0.0298 (8)	0.0043 (7)	0.0068 (6)	0.0027 (7)
C155	0.0244 (7)	0.0438 (9)	0.0302 (8)	0.0010 (7)	0.0102 (6)	0.0054 (7)
C200	0.0191 (6)	0.0251 (7)	0.0229 (7)	−0.0027 (5)	0.0059 (5)	−0.0027 (6)
C201	0.0253 (7)	0.0268 (8)	0.0345 (8)	−0.0044 (6)	0.0120 (6)	−0.0058 (6)
C202	0.0266 (8)	0.0413 (9)	0.0362 (8)	−0.0023 (7)	0.0099 (6)	−0.0164 (7)
C203	0.0267 (8)	0.0623 (11)	0.0223 (7)	−0.0055 (7)	0.0087 (6)	−0.0093 (7)
C204	0.0322 (8)	0.0495 (10)	0.0218 (7)	−0.0082 (7)	0.0075 (6)	0.0026 (7)
C205	0.0291 (7)	0.0294 (8)	0.0221 (7)	−0.0046 (6)	0.0055 (6)	0.0006 (6)
O1	0.0250 (5)	0.0272 (5)	0.0351 (6)	−0.0047 (4)	0.0080 (4)	0.0000 (4)
O2	0.0385 (6)	0.0351 (6)	0.0325 (6)	−0.0149 (5)	0.0119 (5)	−0.0018 (5)

### Geometric parameters (Å, º)

**Table:**

C1—N21	1.3755 (18)	C51—O2	1.4685 (19)
C1—C20	1.3958 (19)	C51—C54	1.514 (3)
C1—C2	1.4317 (19)	C51—C55	1.520 (3)
C2—C3	1.358 (2)	C52—H52A	0.9800
C2—H2	0.9500	C52—H52B	0.9800
C3—C4	1.4382 (19)	C52—H52C	0.9800
C3—H3	0.9500	C53—H53A	0.9800
C4—N21	1.3704 (17)	C53—H53B	0.9800
C4—C5	1.4033 (19)	C53—H53C	0.9800
C5—C6	1.413 (2)	C54—H54A	0.9800
C5—B1	1.573 (2)	C54—H54B	0.9800
C6—N22	1.3641 (18)	C54—H54C	0.9800
C6—C7	1.4549 (19)	C55—H55A	0.9800
C7—C8	1.340 (2)	C55—H55B	0.9800
C7—H7	0.9500	C55—H55C	0.9800
C8—C9	1.4527 (19)	C100—C105	1.393 (2)
C8—H8	0.9500	C100—C101	1.398 (2)
C9—N22	1.3781 (18)	C101—C102	1.389 (2)
C9—C10	1.399 (2)	C101—H101	0.9500
C10—C11	1.4050 (19)	C102—C103	1.387 (2)
C10—C100	1.5007 (19)	C102—H102	0.9500
C11—N23	1.3678 (17)	C103—C104	1.387 (2)
C11—C12	1.428 (2)	C103—H103	0.9500
C12—C13	1.356 (2)	C104—C105	1.390 (2)
C12—H12	0.9500	C104—H104	0.9500
C13—C14	1.428 (2)	C105—H105	0.9500
C13—H13	0.9500	C150—C151	1.385 (2)
C14—N23	1.3734 (18)	C150—C155	1.385 (2)
C14—C15	1.3967 (19)	C151—C152	1.386 (2)
C15—C16	1.405 (2)	C151—H151	0.9500
C15—C150	1.4947 (19)	C152—C153	1.372 (3)
C16—N24	1.3719 (18)	C152—H152	0.9500
C16—C17	1.4454 (19)	C153—C154	1.370 (3)
C17—C18	1.348 (2)	C153—H153	0.9500
C17—H17	0.9500	C154—C155	1.391 (2)
C18—C19	1.4453 (19)	C154—H154	0.9500
C18—H18	0.9500	C155—H155	0.9500
C19—N24	1.3639 (18)	C200—C201	1.394 (2)
C19—C20	1.4151 (19)	C200—C205	1.399 (2)
C20—C200	1.4903 (19)	C201—C202	1.389 (2)
N21—H21	0.8800	C201—H201	0.9500
N23—H23	0.8800	C202—C203	1.382 (3)
B1—O2	1.361 (2)	C202—H202	0.9500
B1—O1	1.368 (2)	C203—C204	1.382 (3)
C50—O1	1.4690 (18)	C203—H203	0.9500
C50—C52	1.509 (2)	C204—C205	1.383 (2)
C50—C53	1.509 (2)	C204—H204	0.9500
C50—C51	1.551 (2)	C205—H205	0.9500
			
N21—C1—C20	125.69 (12)	C55—C51—C50	112.90 (17)
N21—C1—C2	106.93 (11)	C50—C52—H52A	109.5
C20—C1—C2	127.31 (13)	C50—C52—H52B	109.5
C3—C2—C1	107.97 (12)	H52A—C52—H52B	109.5
C3—C2—H2	126.0	C50—C52—H52C	109.5
C1—C2—H2	126.0	H52A—C52—H52C	109.5
C2—C3—C4	108.31 (12)	H52B—C52—H52C	109.5
C2—C3—H3	125.8	C50—C53—H53A	109.5
C4—C3—H3	125.8	C50—C53—H53B	109.5
N21—C4—C5	126.32 (12)	H53A—C53—H53B	109.5
N21—C4—C3	106.57 (11)	C50—C53—H53C	109.5
C5—C4—C3	127.10 (12)	H53A—C53—H53C	109.5
C4—C5—C6	124.40 (13)	H53B—C53—H53C	109.5
C4—C5—B1	117.32 (12)	C51—C54—H54A	109.5
C6—C5—B1	118.28 (12)	C51—C54—H54B	109.5
N22—C6—C5	127.11 (12)	H54A—C54—H54B	109.5
N22—C6—C7	109.88 (12)	C51—C54—H54C	109.5
C5—C6—C7	122.68 (13)	H54A—C54—H54C	109.5
C8—C7—C6	107.20 (12)	H54B—C54—H54C	109.5
C8—C7—H7	126.4	C51—C55—H55A	109.5
C6—C7—H7	126.4	C51—C55—H55B	109.5
C7—C8—C9	106.89 (12)	H55A—C55—H55B	109.5
C7—C8—H8	126.6	C51—C55—H55C	109.5
C9—C8—H8	126.6	H55A—C55—H55C	109.5
N22—C9—C10	126.00 (12)	H55B—C55—H55C	109.5
N22—C9—C8	109.74 (12)	C105—C100—C101	118.56 (13)
C10—C9—C8	124.26 (12)	C105—C100—C10	121.17 (13)
C9—C10—C11	124.82 (12)	C101—C100—C10	120.22 (13)
C9—C10—C100	119.77 (12)	C102—C101—C100	120.81 (14)
C11—C10—C100	115.40 (12)	C102—C101—H101	119.6
N23—C11—C10	126.31 (13)	C100—C101—H101	119.6
N23—C11—C12	107.04 (12)	C103—C102—C101	119.94 (14)
C10—C11—C12	126.57 (13)	C103—C102—H102	120.0
C13—C12—C11	108.37 (13)	C101—C102—H102	120.0
C13—C12—H12	125.8	C102—C103—C104	119.86 (14)
C11—C12—H12	125.8	C102—C103—H103	120.1
C12—C13—C14	107.71 (13)	C104—C103—H103	120.1
C12—C13—H13	126.1	C103—C104—C105	120.14 (14)
C14—C13—H13	126.1	C103—C104—H104	119.9
N23—C14—C15	125.50 (12)	C105—C104—H104	119.9
N23—C14—C13	107.25 (12)	C104—C105—C100	120.69 (14)
C15—C14—C13	127.24 (13)	C104—C105—H105	119.7
C14—C15—C16	125.32 (13)	C100—C105—H105	119.7
C14—C15—C150	117.61 (12)	C151—C150—C155	118.59 (14)
C16—C15—C150	117.06 (12)	C151—C150—C15	120.70 (13)
N24—C16—C15	126.20 (12)	C155—C150—C15	120.65 (13)
N24—C16—C17	110.36 (12)	C150—C151—C152	120.19 (17)
C15—C16—C17	123.44 (12)	C150—C151—H151	119.9
C18—C17—C16	106.57 (12)	C152—C151—H151	119.9
C18—C17—H17	126.7	C153—C152—C151	120.83 (18)
C16—C17—H17	126.7	C153—C152—H152	119.6
C17—C18—C19	106.92 (12)	C151—C152—H152	119.6
C17—C18—H18	126.5	C154—C153—C152	119.55 (15)
C19—C18—H18	126.5	C154—C153—H153	120.2
N24—C19—C20	126.72 (12)	C152—C153—H153	120.2
N24—C19—C18	110.41 (12)	C153—C154—C155	120.11 (15)
C20—C19—C18	122.80 (12)	C153—C154—H154	119.9
C1—C20—C19	124.57 (12)	C155—C154—H154	119.9
C1—C20—C200	119.18 (12)	C150—C155—C154	120.73 (15)
C19—C20—C200	116.19 (12)	C150—C155—H155	119.6
C4—N21—C1	110.18 (11)	C154—C155—H155	119.6
C4—N21—H21	124.9	C201—C200—C205	118.42 (13)
C1—N21—H21	124.9	C201—C200—C20	122.32 (13)
C6—N22—C9	106.23 (11)	C205—C200—C20	119.13 (13)
C11—N23—C14	109.64 (11)	C202—C201—C200	120.45 (14)
C11—N23—H23	125.2	C202—C201—H201	119.8
C14—N23—H23	125.2	C200—C201—H201	119.8
C19—N24—C16	105.71 (11)	C203—C202—C201	120.33 (15)
O2—B1—O1	113.15 (13)	C203—C202—H202	119.8
O2—B1—C5	122.75 (13)	C201—C202—H202	119.8
O1—B1—C5	124.06 (13)	C202—C203—C204	119.86 (14)
O1—C50—C52	105.50 (12)	C202—C203—H203	120.1
O1—C50—C53	109.19 (13)	C204—C203—H203	120.1
C52—C50—C53	110.02 (14)	C203—C204—C205	120.11 (15)
O1—C50—C51	102.03 (11)	C203—C204—H204	119.9
C52—C50—C51	114.05 (15)	C205—C204—H204	119.9
C53—C50—C51	115.20 (14)	C204—C205—C200	120.80 (15)
O2—C51—C54	108.07 (16)	C204—C205—H205	119.6
O2—C51—C55	106.34 (14)	C200—C205—H205	119.6
C54—C51—C55	111.76 (18)	B1—O1—C50	107.07 (12)
O2—C51—C50	102.37 (11)	B1—O2—C51	107.33 (12)
C54—C51—C50	114.50 (15)		
			
N21—C1—C2—C3	1.93 (15)	C20—C19—N24—C16	−178.05 (13)
C20—C1—C2—C3	−175.19 (13)	C18—C19—N24—C16	−1.13 (15)
C1—C2—C3—C4	−1.34 (16)	C15—C16—N24—C19	−178.43 (13)
C2—C3—C4—N21	0.26 (15)	C17—C16—N24—C19	1.58 (15)
C2—C3—C4—C5	−178.83 (13)	C4—C5—B1—O2	−134.75 (15)
N21—C4—C5—C6	−9.8 (2)	C6—C5—B1—O2	44.7 (2)
C3—C4—C5—C6	169.07 (13)	C4—C5—B1—O1	42.9 (2)
N21—C4—C5—B1	169.59 (13)	C6—C5—B1—O1	−137.66 (14)
C3—C4—C5—B1	−11.5 (2)	O1—C50—C51—O2	27.64 (15)
C4—C5—C6—N22	7.2 (2)	C52—C50—C51—O2	−85.58 (15)
B1—C5—C6—N22	−172.24 (13)	C53—C50—C51—O2	145.78 (14)
C4—C5—C6—C7	−165.55 (13)	O1—C50—C51—C54	144.32 (16)
B1—C5—C6—C7	15.0 (2)	C52—C50—C51—C54	31.1 (2)
N22—C6—C7—C8	−1.82 (17)	C53—C50—C51—C54	−97.5 (2)
C5—C6—C7—C8	172.03 (13)	O1—C50—C51—C55	−86.27 (16)
C6—C7—C8—C9	2.27 (17)	C52—C50—C51—C55	160.50 (15)
C7—C8—C9—N22	−2.08 (17)	C53—C50—C51—C55	31.9 (2)
C7—C8—C9—C10	177.52 (14)	C9—C10—C100—C105	−65.26 (18)
N22—C9—C10—C11	2.9 (2)	C11—C10—C100—C105	113.43 (15)
C8—C9—C10—C11	−176.60 (13)	C9—C10—C100—C101	117.51 (15)
N22—C9—C10—C100	−178.50 (12)	C11—C10—C100—C101	−63.80 (17)
C8—C9—C10—C100	2.0 (2)	C105—C100—C101—C102	−0.2 (2)
C9—C10—C11—N23	−6.4 (2)	C10—C100—C101—C102	177.08 (13)
C100—C10—C11—N23	175.03 (12)	C100—C101—C102—C103	0.0 (2)
C9—C10—C11—C12	169.88 (14)	C101—C102—C103—C104	0.3 (2)
C100—C10—C11—C12	−8.7 (2)	C102—C103—C104—C105	−0.3 (2)
N23—C11—C12—C13	0.46 (17)	C103—C104—C105—C100	0.0 (2)
C10—C11—C12—C13	−176.36 (14)	C101—C100—C105—C104	0.3 (2)
C11—C12—C13—C14	−0.79 (17)	C10—C100—C105—C104	−177.03 (13)
C12—C13—C14—N23	0.82 (16)	C14—C15—C150—C151	105.95 (18)
C12—C13—C14—C15	−178.41 (14)	C16—C15—C150—C151	−73.76 (19)
N23—C14—C15—C16	−2.4 (2)	C14—C15—C150—C155	−77.00 (18)
C13—C14—C15—C16	176.65 (14)	C16—C15—C150—C155	103.29 (16)
N23—C14—C15—C150	177.87 (13)	C155—C150—C151—C152	0.0 (3)
C13—C14—C15—C150	−3.0 (2)	C15—C150—C151—C152	177.07 (18)
C14—C15—C16—N24	−2.8 (2)	C150—C151—C152—C153	−0.5 (3)
C150—C15—C16—N24	176.88 (13)	C151—C152—C153—C154	0.5 (3)
C14—C15—C16—C17	177.18 (14)	C152—C153—C154—C155	0.1 (3)
C150—C15—C16—C17	−3.1 (2)	C151—C150—C155—C154	0.6 (2)
N24—C16—C17—C18	−1.47 (17)	C15—C150—C155—C154	−176.50 (14)
C15—C16—C17—C18	178.55 (14)	C153—C154—C155—C150	−0.7 (3)
C16—C17—C18—C19	0.71 (17)	C1—C20—C200—C201	60.41 (19)
C17—C18—C19—N24	0.24 (17)	C19—C20—C200—C201	−117.00 (15)
C17—C18—C19—C20	177.30 (13)	C1—C20—C200—C205	−123.79 (14)
N21—C1—C20—C19	−0.2 (2)	C19—C20—C200—C205	58.80 (17)
C2—C1—C20—C19	176.38 (13)	C205—C200—C201—C202	−1.1 (2)
N21—C1—C20—C200	−177.40 (12)	C20—C200—C201—C202	174.77 (13)
C2—C1—C20—C200	−0.8 (2)	C200—C201—C202—C203	−0.4 (2)
N24—C19—C20—C1	10.7 (2)	C201—C202—C203—C204	1.1 (2)
C18—C19—C20—C1	−165.86 (13)	C202—C203—C204—C205	−0.2 (2)
N24—C19—C20—C200	−172.05 (13)	C203—C204—C205—C200	−1.4 (2)
C18—C19—C20—C200	11.4 (2)	C201—C200—C205—C204	2.0 (2)
C5—C4—N21—C1	−179.92 (13)	C20—C200—C205—C204	−174.01 (13)
C3—C4—N21—C1	0.98 (15)	O2—B1—O1—C50	11.02 (16)
C20—C1—N21—C4	175.39 (13)	C5—B1—O1—C50	−166.80 (13)
C2—C1—N21—C4	−1.79 (15)	C52—C50—O1—B1	95.52 (14)
C5—C6—N22—C9	−173.02 (13)	C53—C50—O1—B1	−146.28 (13)
C7—C6—N22—C9	0.49 (15)	C51—C50—O1—B1	−23.93 (15)
C10—C9—N22—C6	−178.67 (13)	O1—B1—O2—C51	8.13 (17)
C8—C9—N22—C6	0.92 (15)	C5—B1—O2—C51	−174.01 (13)
C10—C11—N23—C14	176.90 (13)	C54—C51—O2—B1	−143.58 (15)
C12—C11—N23—C14	0.06 (15)	C55—C51—O2—B1	96.28 (17)
C15—C14—N23—C11	178.71 (13)	C50—C51—O2—B1	−22.37 (16)
C13—C14—N23—C11	−0.54 (15)		

### Hydrogen-bond geometry (Å, º)

Cg1 is the centroid of the C100–C105 phenyl ring.

**Table:**

*D*—H···*A*	*D*—H	H···*A*	*D*···*A*	*D*—H···*A*
N21—H21···N24	0.88	2.35	2.9084 (16)	122
N21—H21···N22	0.88	2.38	2.9266 (16)	121
N23—H23···N24	0.88	2.34	2.8978 (16)	121
N23—H23···N22	0.88	2.35	2.8986 (16)	120
C153—H153···*Cg*1^i^	0.95	2.58	3.488 (2)	160

Symmetry code: (i) *x*−1, *y*, *z*.
