# Supplementary figures and images for: Crystal structure of 5,10,15-triphenyl-20-(4,4,5,5-tetra­methyl-1,3,2-dioxaborolan-2-yl)porphyrin
Source: Acta Crystallogr Sect E Struct Rep Online. 2014 Sep 6;70(Pt 10):o1085–6. doi: 10.1107/S1600536814019680 (PMC4257212; doi:10.1107/S1600536814019680)

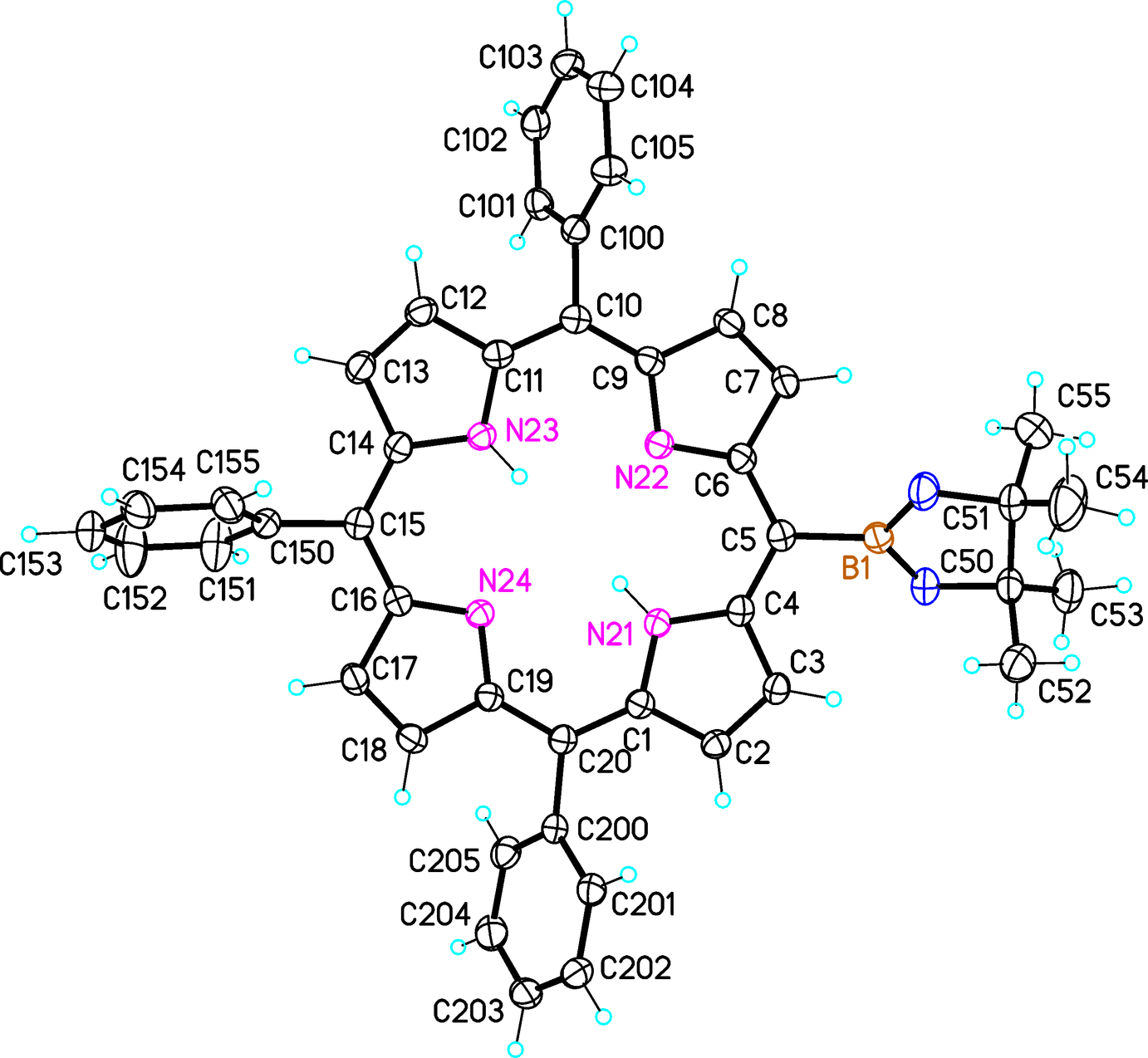

Supplement: Supplementary file 3 [file e-70-o1085-fig1.tif]

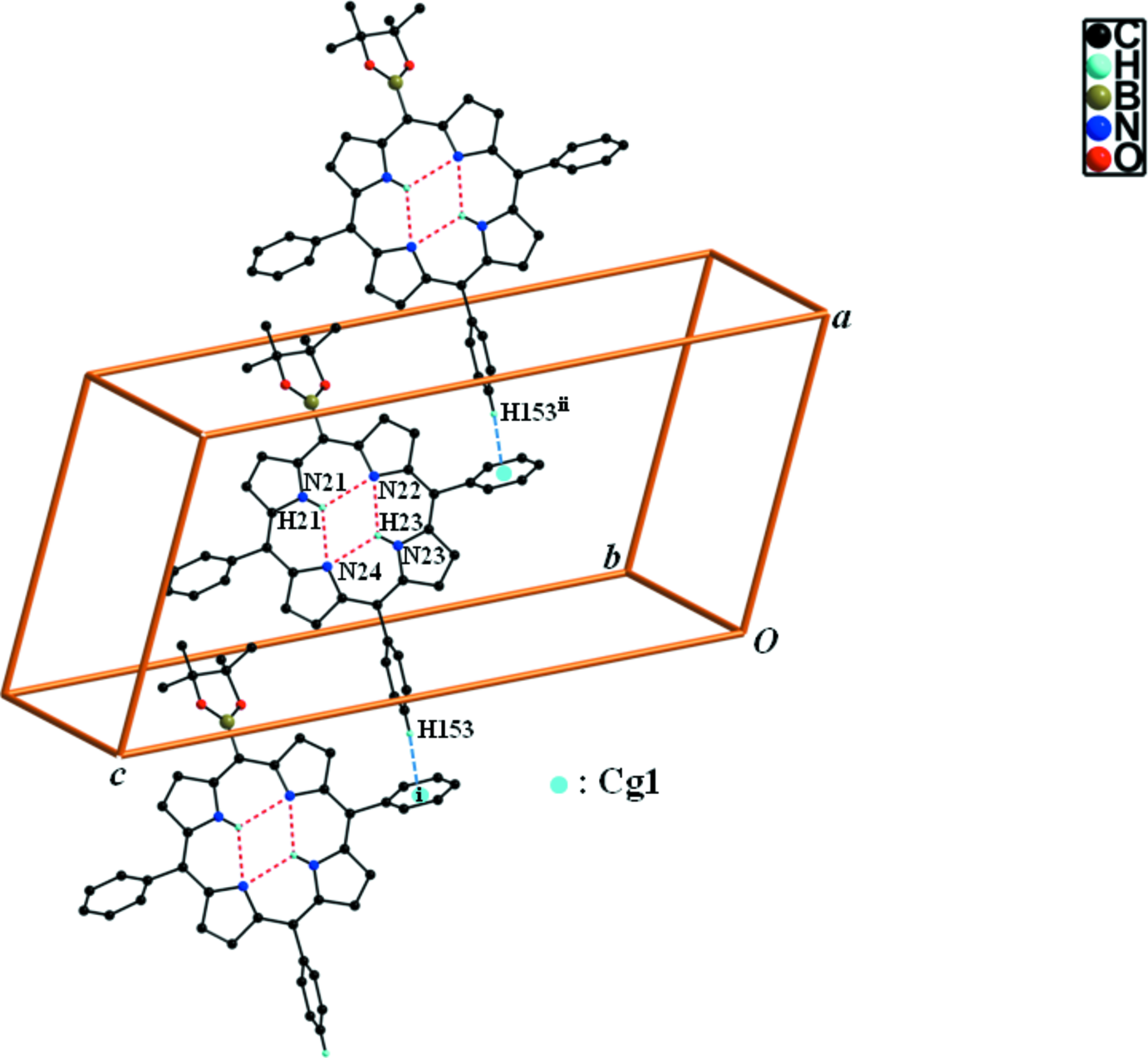

Supplement: Supplementary file 4 [file e-70-o1085-fig2.tif]
